# The Role of Targeted Therapy in Metastatic Renal Cell Carcinoma

**DOI:** 10.1100/tsw.2007.149

**Published:** 2007-03-02

**Authors:** Jaya Unnithan, Brian I. Rini

**Affiliations:** Department of Solid Tumor Oncology, Cleveland Clinic Taussig Cancer Center, Cleveland, OH, USA

**Keywords:** renal cell carcinoma, VHL, VEGF, bevacizumab, sorafenib, sunitinib, temsirolimus

## Abstract

Renal cell carcinoma (RCC) is a highly vascular tumor in which a growing understanding of disease biology has been translated into clinically active systemic therapies. The most clinically developed targeted therapies in advanced RCC are those that target the vascular endothelial growth factor (VEGF) ligand or receptor (VEGFR) and therapy directed against the mammalian target of rapamycin (mTOR). Sutent and sorafenib are orally available inhibitors of the VEGFR and platelet derived growth factor receptor (PDGFR). Temsirolimus is an mTOR inhibitor that leads to G_1_ cell cycle arrest and may affect VEGF production. This article briefly describes the biological pathways involved in the development of RCC and the results of clinical trials using targeted therapy in metastatic RCC.

## INTRODUCTION

The estimated number of new patients with renal cell carcinoma (RCC) in 2006 is 38,890[1]. Renal cell carcinoma is a highly vascular tumor, which is resistant to most forms of standard cancer treatment including chemotherapy and radiotherapy. Immunotherapy has historically been used to treat metastatic RCC. Interferon (IFN) produces a response rate of 15-20% with a modest survival advantage over inactive therapy[2,3]. Interleukin-2 (IL-2) can be given in low doses subcutaneously or as high-dose intravenously. Response rates of 15-20% are observed, with a small proportion (4-5%) achieving a durable complete response[4,5,6]. During the past decade, there has been an explosive understanding of the molecular mechanisms involved in RCC associated with the von Hippel-Lindau (VHL) syndrome. This molecular pathway is also altered in clear cell sporadic RCC, and thus therapeutic targets have emerged. Clinical targeting of various aspects of this pathway has resulted in substantial gains in the clinical treatment of metastatic RCC.

## The VHL pathway in RCC

VHL is an autosomal dominant hereditary cancer syndrome characterized by the development of renal cysts, retinal and cerebellar hemangioblastomas, pancreatic cysts and carcinomas and renal cell carcinoma. In the early 1990s the *VHL* tumor suppressor gene was identified and mapped to chromosome 3p25[7]. Somatic inactivation of both copies of the *VHL* gene by mutation, deletion or methylation has been identified in up to 60% of sporadic clear cell renal cell carcinomas[8,9,10].

The most important function of the protein product of the *VHL* gene (pVHL) is to bind to target proteins as an adapter molecule in the multi subunit ubiquitin ligase complex. The most important pVHL target protein is the α subunit of the hypoxia inducible factor 1 (HIF-1). Under normoxic conditions, HIF-1 α is hydroxylated and forms a complex with the VHL protein complex. Under hypoxia, HIF is not hydroxylated and therefore does not interact with VHL. A similar situation occurs when there is a loss of function of the VHL gene as in RCC. In this circumstance, the HIF-1α transcription factor accumulates. HIF-1 α binds with HIF-1 β and the complex leads to transcription of a variety of genes responsible for the high expression of VEGF[11,12] . Other growth factors that are over expressed in renal cell carcinomas as a result of VHL pathway activation are PDGF and TGF-α. These mitogenic growth factors bind to the receptor tyrosine kinases on the cell membranes of RCC cells and activate downstream signal transduction cascades, which lead to cell cycle progression and proliferation. The importance of VHL pathway and angiogenesis in clear cell RCC led to clinical trials utilizing angiogenesis inhibitors in the management of metastatic renal cell carcinoma.

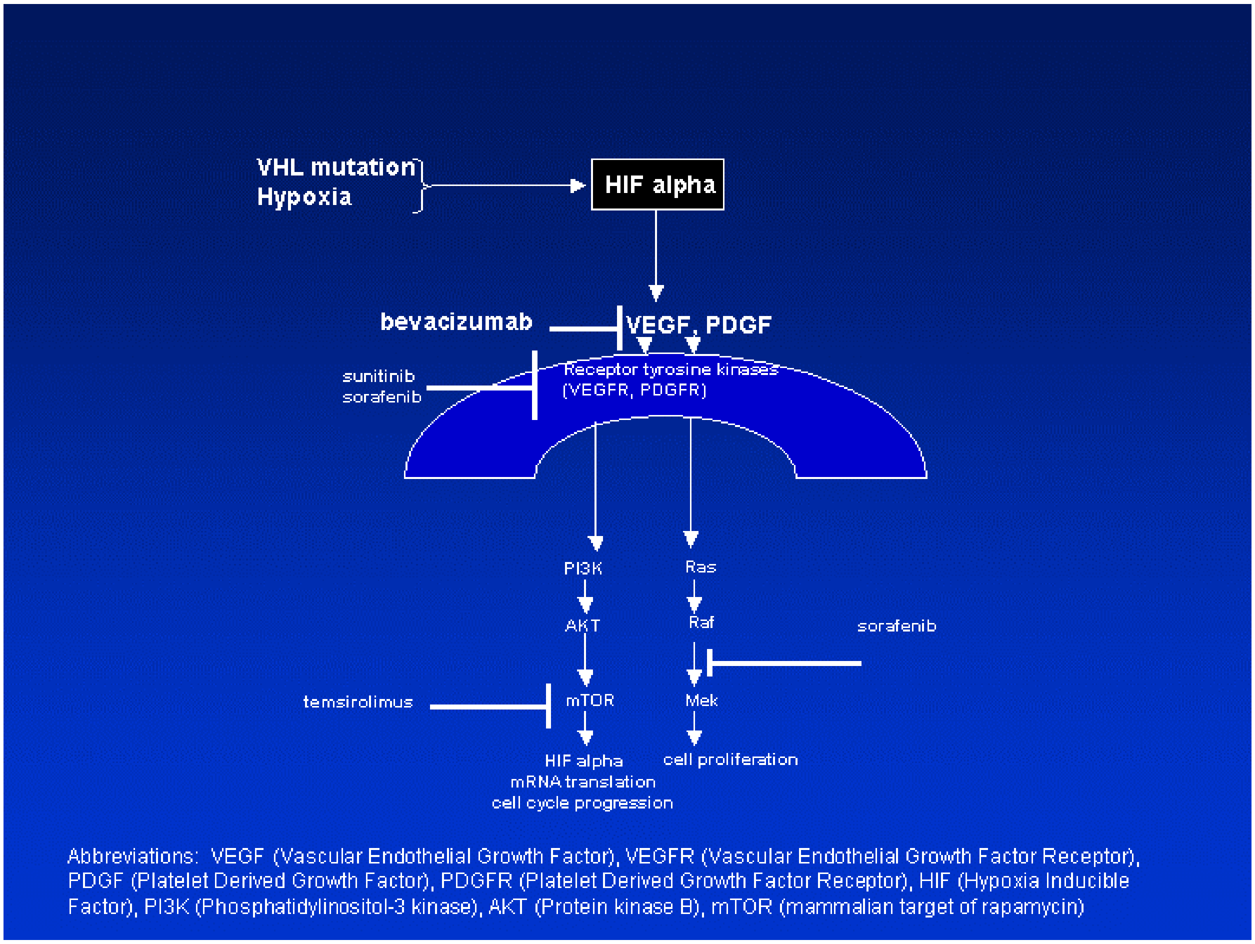

## Bevacizumab (Avastin®)

Bevacizumab is a humanized monoclonal antibody to VEGF (the murine parent antibody is muMAB A.4.6.1). In 1997 Presta et al.[13] demonstrated the effects of a humanized VEGF inhibitor and showed that it inhibits VEGF-induced proliferation of endothelial cells *in vitro* and tumor growth *in vivo* with potency and efficacy very similar to those of the murine monoclonal antibody. The clinical effect was studied in a phase II RCC trial that randomized 116 patients to receive either low dose (n=37) or high dose bevacizumab (n=39) or placebo (n=40)[14]. The progression free survival in the group treated with high dose bevacizumab was significantly longer than the group treated with placebo (4.8 months versus 2.5 months, p=0.001). There was a marginally significant improvement when the low dose group was compared to placebo (p=0.053). Hypertension and proteinuria were the most common side effects most notable in the high dose bevacizumab arm. Bevacizumab has also been studied in combination with the epidermal growth factor receptor (EGFR) inhibitor erlotinib[15] in which 63 patients were treated and a response rate of 25% was observed, with additional patients experiencing minor tumor shrinkage. A subsequent randomized trial investigated the combination of bevacizumab and erlotinib and was compared with bevacizumab alone in randomized phase II trial[16]. The progression free survival in the group that received bevacizumab and erlotinib was 9.9 months vs. 8.5 months in the group that received only bevacizumab (p=n.s.). This trial demonstrated a substantial PFS for bevacizumab in untreated metastatic RCC patients, and also that adding erlotinib is not of benefit. Two randomized phase III trials have also been completed which randomized untreated metastatic clear cell RCC patients to IFN alone or IFN plus bevacizumab and were powered to investigate an overall survival benefit. Results from these trials will add to the data regarding the effect of bevacizumab in metastatic RCC.

## Sorafenib (Nexavar®)

Sorafenib is an oral multi-kinase inhibitor that targets Raf kinases (Raf-1, wild-type B-Raf, and b-Raf V600E), in addition to receptor tyrosine kinases associated with angiogenesis such as VEGFR and PDGFR[17,18]. The activity of sorafenib on metastatic RCC was demonstrated in phase I studies using a continuous dosage of 400mg BID[19]. Dose limiting toxicities observed were grade 3 diarrhea, hand-foot syndrome and fatigue. Sorafenib was then compared with placebo in a phase II randomized discontinuation study (n=202)[20]. All patients received twelve weeks of sorafenib, and then patients with tumor burden change within 25% of baseline measurements were randomized to placebo or continuation of sorafenib. The median progression free survival was significantly longer in the sorafenib group (24 weeks versus 6 weeks in the placebo group, p=0.0087). The most common adverse events were fatigue, rash / desquamation, hand foot skin reaction and diarrhea. In a phase III trial[21] patients who received one prior systemic therapy were randomized to receive either continuous sorafenib 400 mg bid or matched placebo with best supportive care (BSC). The secondary end point was PFS, which was reported after 363 events. The median PFS was significantly longer in the sorafenib treated group (24 weeks) compared to placebo (12 weeks; p<0.000001). Based on these data, sorafenib was approved by the FDA for the treatment of advanced renal cell carcinoma in December 2005.

The combination of sorafenib with other agents is also being investigated in metastatic RCC. The combination of sorafenib and IFN alpha was investigated in two phase II studies[22,23]. These studies demonstrated response rates of 42% and 19%. Toxicity was as expected for each agent, with a notable low incidence of hand foot syndrome. Further investigation is ongoing. A phase I/II trial with bevacizumab and sorafenib in metastatic RCC is also currently ongoing. Limited data is available thus far, but suggests less than full doses of both drugs are tolerable[24].

## Sunitinib (Sutent®)

Sunitinib is a potent orally active inhibitor of multiple protein tyrosine kinases including VEGFR and PDGFR. Two multi-center phase II trials were undertaken to evaluate safety and efficacy of sunitinib dosed as 50mg daily for 28 days, followed by 14 days of rest in patients with advanced metastatic RCC [25,26]. The first trial was conducted on 63 metastatic RCC patients who failed prior cytokine-based therapy. The overall response rate per RECIST criteria was 40%, a substantial improvement over historical standards. To confirm the results of this trial, a second open label multi center trial was initiated treating 106 patients with cytokine-refractory clear cell RCC and demonstrated a response rate of 43%. The combined analysis of both phase II trials revealed a response rate of 42% and a PFS of 8.2 months. These studies demonstrate that sunitinib is an effective second line therapy in cytokine refractory RCC. Based on these data, sunitinib was FDA approved for the treatment of advanced RCC in January 2006.

Continuous dosing of sunitinib 37.5 mg daily has also been investigated in a phase II multi center study, in which patients were randomized to receive 37.5 mg of sunitinib either in the morning or in the evening[27]. Preliminary data demonstrates a response rate of 15% and a median PFS of 8.3 months. The response rate data is immature, and thus comparisons to the interrupted sunitinib dosing schedule can not be made. It may be that higher, intermittent doses are required for a higher response rate, but that PFS is identical for either schedule. Further follow up is needed.

In an ongoing phase III trial in untreated metastatic RCC (n=750)[28], sunitinib is being compared with IFN to explore the possibility of front line usage of this potent agent. Preliminary data presented indicate a statistically significant difference in the progression free survival 47.3 vs.24.9 weeks ($p<0.000001$). The objective response rate by third-party independent review was 24.8% (95% CI 19.7, 30.5) for sunitinib vs. 4.9% (95% CI 2.7, 8.1) for IFN-α ($p<0.000001$). This study demonstrated the superior efficacy of sunitinib over IFN in the first line treatment of metastatic RCC. Overall survival data is not presently available.

## The mTOR Pathway

Mammalian target of rapamycin (mTOR) is a protein kinase of the phosphatidylinositol 3-kinase (PI3K)/Akt signaling pathway, which plays an important role in multiple intracellular signaling pathways leading to tumor formation and growth. mTOR phosphorylation initiates mRNA translation and subsequent translation of proteins involved in cell growth and differentiation including HIF- alpha, VEGF and PDGF.

## Temsirolimus

Temsirolimus is an inhibitor of mTOR. Temsirolimus leads to cell cycle arrest in G1 phase in pre clinical studies. An initial phase II trial[29] in metastatic RCC randomized patients to one of three dose levels of temsirolimus: 25mg, 75mg or 125 mg. The objective response rate for the entire cohort was 7%, and the median time to progression was 5.8 months. The toxicities reported were maculopapular rash (76%), mucositis (70%), asthenia (50%), and nausea (43%). The most frequently occurring grade 3 or 4 adverse events were hyperglycemia (17%), hypophosphatemia (13%), anemia (9%), and hypertriglyceridemia (6%). A retrospective analysis found that patients with multiple adverse risk factors had a more favorable outcome than historic controls. Thus, a subsequent phase III trial randomized untreated metastatic RCC patients with 3 or more of 6 adverse risk features to IFN alone, temsirolimus alone or IFN plus temsirolimus[30]. Adverse risk features includes LDH > 1.5x upper limit of normal, hemoglobin < lower limit of normal, corrected serum calcium > 10 mg/dL, time from diagnosis of RCC to treatment of > 1 year, Karnofsky performance status 60-70% and multiple organ sites of metastases. The median survival was highest in the temsirolimus arm (10.9 months) compared to 7.3 months in the IFN monotherapy arm

(p=0.0069) and 8.4 months in the combined temsirolimus plus IFN arm. The grade 3 adverse events were asthenia, anemia and dyspnea; anemia and asthenia were higher among patients who received IFN-containing regimens. Temsirolimus is being further investigated in metatstatic RCC in combination with agents that target the VEGF pathway based on the preclinical evidence that the two pathways converge to regulate HIF.

**TABLE 1**
**Summary of select phase II /III trials using targeted therapy in metastatic RCC**

| Agent | Trial design | Results | Major toxicities |
| --- | --- | --- | --- |
| Bevacizumab | Randomized double blind phase II (cytokine-refractory)<br>Low or high dose bevacizumab vs. placebo<br>n=116[14] | PFS:4.8 months with high dose (vs. 2.5 months with placebo); p=0.0001 | Hypertension, proteinuria |
| | Single arm phase II<br>Bevacizumab + erlotinib<br>n=63[15] | ORR: 25%; PFS 11 months | Hypertension, rash, diarrhea |
| | Randomized phase II (untreated pts.)<br>Bevacizumab/ erlotinib vs. bevacizumab/placebo n=104[16] | ORR: 13% (vs.14% with bevacizumab + erlotinib)<br>PFS: 8.5 months with bevacizumab monotherapy (vs. 9.9 months with combination; p=n.s.) | Hypertension, rash, diarrhea, bleeding |
| Sorafenib | Phase II randomized discontinuation trial<br>n=202[20] | PFS:50% at 24 weeks (vs.18% with placebo) p=0.0077 | Hand foot syndrome, skin rash/desquamation,<br>hypertension, fatigue |
| | Phase III randomized trial (cytokine-refractory)<br>Sorafenib vs. placebo<br>n=611[21] | PFS: 24 vs. 12 weeks ($p$<0.000001) | Hand foot syndrome, rash/ desquamation, fatigue,<br>anorexia |
| | Single arm phase II<br>Sorafenib + IFN<br>n=31[22] | ORR: 37% | NR |
| | Single arm phase II<br>Sorafenib + IFN<br>n=67[23] | ORR: 19% | Fatigue, anorexia, diarrhea, nausea, fever, anemia, leukopenia |
| | Single arm phase I<br>Sorafenib + bevacizumab<br>n=18[24] | OR/PFS: NR | Hypertension, Hand foot syndrome, proteinuria |

**TABLE 1 cont.**

| | | | |
|---|---|---|---|
| Sunitinib | Single arm phase II trials<br>n=63 and 106[25,26] | PFS: 8.2 months, ORR:42% | Fatigue, hand foot syndrome, diarrhea,<br>cytopenias, mucositis |
| | Phase III randomized trial (untreated pts.)<br>Sunitinib vs. .IFN<br>n=750 | PFS:11 months (vs.5 months with IFN); p<0.000001<br><br>ORR: 31% (vs.6% with IFN)<br>p<0.000001 | |
| Temsirolimus | Phase II random assignment to one of the 3 doses: 25, 75 or 250 mg of temsirolimus weekly<br>n-=111[29] | ORR: 7%, PFS: 5.8 months | Rash, mucositis, asthenia, nausea<br>hyperglycemia, hypophosphatemia, hypertriglyceridemia |
| | Phase III randomized three arm<br>temsirolimus with IFN, vs. IFN alone and temsirolimus alone, n=626[30] | PFS: 3.7 months for monotherapy arm (vs.1.9 months for IFN)<br><br>OS: 10.9 months (vs. 7.3 months for IFN); p=0.0069 | Asthenia, anemia, dyspnea |

## DISCUSSION

Renal cell carcinoma has historically been a treatment resistant tumor. Association of renal cell carcinoma with inherited VHL syndrome and the subsequent elucidation of relevant biological pathways in clear cell RCC have changed the approach to systemic therapy. Drugs targeting the VEGF and related pathways have robust clinical activity. Ongoing clinical trials are exploring multiple options such as combining molecular targets with cytokines and combining targeting agents directed against different proteins in the same or different molecular pathways. The emergence of active agents in RCC has generated several needs within the RCC clinical research arena. One pressing need is a pre-clinical model that can replicate the phenotype of disease and treatment with these drugs to better understand mechanisms of response and resistance. Predictive clinical and molecular markers also require investigation to most appropriately target these agents. In addition, standard CT scans and RECIST criteria are often inadequate to evaluate response and progression with VEGF-targeted therapy. Investigation of newer imaging modalities and evaluation criteria is warranted. A new era of treatment in metastatic RCC has emerged, however, where rational therapeutic targeting has lead to substantial clinical advances.
